# Association between childhood family structure and health-related quality of life at middle age: A longitudinal study of Northern Finland Birth Cohort 1966

**DOI:** 10.1177/14034948241260765

**Published:** 2024-08-06

**Authors:** Heidi Varis, Eveliina Heikkala, Ilona Mikkola, Tanja Nordström, Anja Taanila, Sirkka Keinänen-Kiukaanniemi, Maria Hagnäs

**Affiliations:** 1 Research Unit of Population Health, University of Oulu, Finland; 2 Wellbeing Services, County of Lapland, Rovaniemi, Finland; 3 Medical Research Centre Oulu, Oulu University Hospital and University of Oulu, Finland; 4 Northern Finland Birth Cohorts, Arctic Biobank, Infrastructure for Population Studies, Faculty of Medicine, University of Oulu, Finland; 5 Unit of Primary Care, Oulu University Hospital, Finland; 6 Healthcare and Social Services of Selänne, Pyhäjärvi, Finland

**Keywords:** Childhood family structure, health-related quality of life, offspring, single-parent, longitudinal

## Abstract

**Aims:**

This longitudinal study evaluated the association between childhood family structure and health-related quality of life (HRQoL) at middle age.

**Methods:**

The data on childhood family structure at the age of 14 years (‘two-parent family’, ‘one parent not living at home/no information on father’ and ‘father or mother deceased’) and HRQoL (measured by 15D (15-dimensional)) at the age of 46 were collected from the Northern Finland Birth Cohort 1966 using postal questionnaires. We used the binary logistic regression model to estimate the associations between childhood family structures and the lowest 15D quartile (reference: all other quartiles). The associations were adjusted for offspring mothers’ factors during pregnancy (mothers’ educational and occupational status).

**Results:**

Of the 6375 participants, the offspring belonging to the ‘one parent not living at home/no information on father’ family structure subgroup had higher odds ratio of belonging to the lowest 15D quartile than the offspring of ‘two-parent families’ (adjusted odds ratio (OR) 1.76, 95% confidence interval (CI) 1.31–2.36, p<0.001 for females; adjusted OR 1.86, 95% CI 1.28–2.70, p=0.001 for males). There were no statistically significant associations between the ‘father or mother deceased’ subgroup and the lowest 15D quartile among the offspring.

**Conclusions:**

A single-parent family origin (due to reasons other than parental death) in childhood was significantly associated with impaired HRQoL at middle age. These results provide new perspectives for understanding the long-standing associations on living in a single-parent family.

## Background and aims

Childhood family structure has far-reaching associations with offspring’s health and welfare [1–3]. The two-parent family structure (in which both biological parents live in the same household as their offspring) is associated with a more beneficial impact on the offspring’s psychological general welfare and health than the single-parent family structure [1,4–6]. Owing to increased divorce rates and births outside marriage in the Western world, the number of offspring living in single-parent families and in different family environments is higher today than it was a few decades ago [7,8]. The growing number of households headed by a single parent highlights the importance of research on the childhood family structure and its associations with offspring’s welfare.

Family structure and environment may also play an important role in the offspring’s quality of life. High family affluence, easy communication with parents, and living with both parents have been associated with higher quality of life in adolescence [9]. Children living with two parents report fewer mood problems, pain or problems performing normal activities than children living in other family constellations, which reflects differences in the quality of life in adolescence [10]. Moreover, family-related stressful life events or adverse childhood experiences (including parental separation and parental death) have been associated with poorer physical and psychosocial quality of life among offspring across the lifespan [9,11–15]. This indicates that parental separation or death may also have long-term effects on health-related quality of life (HRQoL) in adulthood.

To date, there is a lack of studies investigating the association between childhood family structure and HRQoL in adulthood. As individuals with impaired HRQoL have a greater need for health care services [16] and are at a higher risk of premature mortality [17], it is essential to identify the early factors associated with decreased levels of HRQoL. Characterizing and considering the potential early factors may alleviate impaired HRQoL and its consequences. In the comprehensive exploration of both physical and psychosocial HRQoL, the 15D (15-dimensional) instrument has shown to be a practical, valid tool. It consists of 15 dimensions: breathing, mental function, speech (communication), vision, mobility, usual activities, vitality, hearing, eating, elimination, sleeping, distress, discomfort and symptoms, sexual activity, and depression [18].

In this study, we investigated the longitudinal association between childhood family structure at the age of 14 years (two-parent *vs*. single-parent family (divided into one parent not living at home, father or mother deceased, and no information on father)) and HRQoL (measured by 15D) at the age of 46, using data on a large population-based birth cohort. We hypothesized that living in a single-parent family in childhood is associated with impaired HRQoL in adulthood.

## Methods

### Study design and study population

The population of this large longitudinal population-based study stems from the Northern Finland Birth Cohort 1966 (NFBC1966), which comprises 96.3% of all live births in the two northernmost provinces in Finland with expected delivery dates in 1966 (initially a total of 12,231 individuals) [19,20]. Data collection was initiated during the 24th gestational week, and follow-up data have been gathered at several time points from birth to midlife. A more precise description of the NFBC1966 has been provided elsewhere [19]. In this study, we used postal questionnaire-based information collected from the participants’ mothers’ pregnancy period and when the participants were aged 14 and 46 years. Data on family structure were gathered when they were 14, HRQoL data and further characteristics data of the offspring when they were 46, and information on confounding factors during the mother’s pregnancy. The final population of the present study totalled 6375 participants (3497 females and 2878 males). The study protocol was approved by the Ethics Committee of the Northern Ostrobothnia Hospital District 94/2011 (12 December 2011). All the study participants (hereafter offspring) provided their written informed consent.

### Family structure

The information on childhood family structure was supplied by the offspring at the age of 14 years using the following statements:1) mother/father is alive, 2) mother/father is deceased, 3) mother/father is alive but not living at home, and 4) no information on father. On the basis of the responses, the variable was categorized as ‘a two-parent family’, ‘one parent not living at home’, ‘father or mother deceased’ and ‘no information on father’. Due to its low number of offspring, the ‘no information on father’ group was combined with the ‘one parent not living at home’ group. The two-parent family was used as the reference.

### HRQoL

HRQoL was estimated at 46 years using the 15D questionnaire, which is a widely used, standardized and validated instrument for evaluating HRQoL in general populations [21,22]. It consists of 15 dimensions: breathing, mental function, speech (communication), vision, mobility, usual activities, vitality, hearing, eating, elimination, sleeping, distress, discomfort and symptoms, sexual activity, and depression. Each dimension is rated by the participant on an ordinal severity scale with five levels (0=no problems, 5=extreme problems/unable), of which individuals select the option that best reflects their current state of health. The dimensions can be used separately or as a single index score measure. The single-index score (hereafter 15D score) of all the dimensions is calculated using a set of population-based utility-of-preference weights and represents overall HRQoL on a scale of 0 to 1 (0 = deceased, 1 = full health) [23]. Each dimension is also scaled in a similar way when assessed separately. A change of more than 0.015 in the 15D score indicates clinical importance, because people can usually identify such a magnitude of improvement or reduction in the 15D score [18,21].

In this study, we divided the 15D sum score into quartiles to study the associations between different childhood family structures and the lowest quartile. All the quartiles except the lowest one were combined and used as the reference in the analyses.

### Confounding factors

The confounding factors were offspring mother’s educational and occupational status during pregnancy. Mother’s educational status during pregnancy was divided into three categories: 1) low 0–4 years, 2) intermediate 5–8 years, and 3) high ⩾ 9 years. Mother’s occupational status during pregnancy was categorized as follows: no occupation (housewife), low social class (unskilled workers, farmers and farmers’ wives) and high social class (professionals and skilled workers).

### Descriptive variables

The following offspring’s factors at the age of 46 were used as characteristics variables: educational level, marital status, number of under-18-years-old children in the household, number of chronic somatic diseases and number of mental health diseases.

Educational level was categorized as follows: 1) basic education or less (under 10 years), 2) secondary education (10–12 years), and 3) tertiary education (over 12 years) [24]. Marital status was categorized into four groups: in a relationship (marriage or cohabitation), unmarried, separated, and widowed. Offspring were distributed into four categories according to the number of children aged under 18 in the household: 0, 1, 2–4, and 5 or more. This was partly based on the grand multiparity definition [25]. The number of chronic somatic diseases variable was formulated by summing the following self-reported diseases (diagnosed by a medical doctor): hypertension, congenital heart disease, heart failure, coronary artery disease/angina pectoris, type 1 diabetes, type 2 diabetes, hypothyroidism, hyperthyroidism, inflammatory bowel disease (Crohn’s disease or ulcerative colitis), psoriasis, increased eye pressure, glaucoma, cataract, macular degeneration, epilepsy, cerebrovascular accident (stroke or apoplexy), cancer (not including skin cancers), fibromyalgia, rheumatoid arthritis, childhood rheumatism, ankylosing spondylitis, other rheumatoid or autoimmune disease, asthma, and emphysema. We also included obesity, defined as body mass index >30 kg/m^2^ (weight and height measurements were primarily based on measured values and secondarily on self-reported values), in the chronic somatic disease sum variable. As a result, the sum variable had a theoretical range of 0–25. We next dichotomized the number of chronic somatic diseases as zero or one versus two or more. The cut-off of two diseases was based on the current definition of multimorbidity [26]. We also assessed the following self-reported mental health diseases diagnosed by a medical doctor: psychosis, depression, other mental disorder, drinking problem, and other psychoactive drug problem. To enable us to study the presence of any or the absence of all of these mental health diseases, we divided the offspring into two categories: mental health disease and no mental health diseases.

### Statistical analysis

The associations between the family structure and the characteristics of the study population were evaluated through cross-tabulation (using Pearson’s chi-squared test or Fisher’s exact test when appropriate), excluding the 15D sum score and 15D dimensions, which were tested using the Mann–Whitney *U*-test. We used maximum likelihood estimation based binary logistic regression to estimate the association between family structure and the lowest HRQoL quartile. We present both the unadjusted and adjusted odds ratios (ORs) with 95% confidence intervals (CIs). To analyse the representativeness of the study sample, differences in the distribution of sex, mother’s educational and mother’s occupational status between the participants and non-participants cohort members were evaluated through cross-tabulation (using Pearson’s chi-squared test).

The analyses were conducted using IBM SPSS Statistics 28 (IBM Corp. Released 2021. IBM SPSS Statistics for Windows, Version 28.0. Armonk, NY: IBM Corp.). All the tests were two-tailed and all *p*-values less than 0.05 were considered statistically significant.

## Results

Table I presents the characteristics of the study population at the age of 46 years for females and Table II presents them for males. The threshold for the lowest 15D quartile was 0.892 for females and 0.909 for males. A total of 2982 females belonged to the ‘two-parent family’ subgroup, 285 to the ‘one parent not living at home/no information on father’ subgroup, and 230 to the ‘father or mother deceased’ subgroup. The respective numbers of males belonging to these groups were 2511, 189 and 178. Among both sexes, the offspring belonging to the ‘one parent not living at home/no information on father’ family structure subgroup were significantly more likely to have two or more chronic somatic diseases than the ‘two-parent family’ offspring (29.5% *vs*. 23.7%, *p*=0.029 for females and 27.1% *vs*. 20.7%, *p*=0.039 for males). In addition, the male offspring of the ‘one parent not living at home/no information on father’ subgroup were more likely to have at least one mental health disease than the male offspring of two-parent families (19.7% *vs*. 12.0%, *p*=0.002). The offspring of the ‘one parent not living at home/no information on father’ subgroup had significantly lower 15D sum scores (0.910 (SD 0.070) *vs*. 0.924 (SD 0.065), *p*<0.001 for females and 0.916 (SD 0.080) *vs*. 0.936 (SD 0.062), *p*=0.008 for males) and were more likely to be in the lowest 15D quartile than those in two-parent families, regardless of their sex (34.0% *vs*. 23.8%, *p*=0.001 for females and 37.6% *vs*. 24.2%, *p*<0.001 for males). The female offspring affected by their father or mother dying during their childhood were significantly more likely to have two or more chronic somatic diseases (30.4% *vs*. 23.7%, *p*=0.021) than the ‘two-parent family’ offspring. There were significant differences (*p* <0.001) between the participants and non-participants, for example, the participants were more often women and the proportion of mothers who were highly educated and had high occupational status was greater (Supplemental material 1 online).

**Table I.** Characteristics of female offspring at the age of 46 years stratified by family structure at the age of 14 years.

	**Two-parent family** *n*= 2982 (85.3 %)		**One parent not living at home/ No information on father** *n*= 285 (8.1 %)			**Father or mother deceased** *n*= 230 (6.6 %)		
	*n*	%/mean (SD)	*n*	%/mean (SD)	*p*	*n*	%/mean (SD)	*p*
**Education level**								
Basic or less	91	3.1	15	5.3	0.130	10	4.4	0.074
Secondary	1960	65.8	185	64.9		163	71.2	
Tertiary	928	31.2	85	29.8		56	24.5	
**Marital status**								
Cohabiting/in relationship	2318	78.0	208	73.0	0.210	169	73.8	0.477
Unmarried	300	10.1	38	13.3		28	12.2	
Divorced/separated	336	11.3	36	12.6		31	13.5	
Widower	19	0.6	<5	1.1		<5	0.4	
**Number of children under 18 years in the household**								
No children	676	25.3	75	29.6	0.166	56	28.0	0.111
One child	822	30.7	84	33.2		72	36.0	
2–4 children	1110	41.5	90	35.6		70	35.0	
Grand multiparity (five or more children)	69	2.6	<5	1.6		<5	1.0	
**Number of chronic somatic diseases**								
No chronic diseases/one chronic disease	2275	76.3	201	70.5	0.029	160	69.6	0.021
Two or more chronic diseases	706	23.7	84	29.5		70	30.4	
**Number of mental health diseases**								
No mental health diseases	2459	82.8	228	80.3	0.292	195	85.9	0.225
One or more mental health diseases	512	17.2	56	19.7		32	14.1	
**15D sum score**	0.924 (0.065)		0.910 (0.070)		<0.001	0.920 (0.058)		0.054
**15D quartiles**								
Lowest quartile (<0.892)	586	23.8	80	34.0	0.001	55	29.4	0.085
Other quartiles	1874	76.2	155	66.0		132	70.6	
**Mother’s educational status (during pregnancy)**								
Low 0–4 years	274	9.3	15	5.4	0.059	34	15.2	0.002
Intermediate 5–8 years	1626	55.3	153	55.0		131	58.5	
High ⩾9 years	1038	35.3	110	39.6		59	26.3	
**Mother’s occupational status (during pregnancy)**								
No occupation	871	29.9	77	28.0	<0.001	76	33.6	0.025
Low social class	1208	41.4	163	59.3		73	32.3	
High social class	837	28.7	35	12.7		77	34.2	

Data are presented as number and percentage or mean and SD (standard deviation) of the population. *N* varies due to missing data. The *p*-value presents the statistical differences between two-parent family and the other family structures in terms of estimated variables, and was evaluated using Pearson’s chi-squared test or Fisher’s exact test, when appropriate, excluding 15D sum score, which was tested with Mann–Whitney *U*-test. The null hypothesis was that the estimated coefficients for the given family structure are not different from those estimated for the two-parent family offspring.

15D: 15-dimensional.

**Table II.** Characteristics of male offspring at the age of 46 years stratified by family structure at the age of 14 years.

	**Two-parent family** *n*= 2511 (87.2%)		**One parent not living at home/No information on father** *n*= 189 (6.6 %)			**Father or mother deceased** *n*= 178 (6.2 %)		
	*n*	%/mean (SD)	*n*	%/mean (SD)	*p*	*n*	%/mean (SD)	*p*
**Education level**								
Basic or less	105	4.2	9	4.8	0.326	17	9.6	0.003
Secondary	1802	71.9	143	76.1		125	70.6	
Tertiary	599	23.9	36	19.1		35	19.8	
**Marital status**								
Cohabiting/in relationship	1976	79.1	134	71.3	0.061	132	74.6	0.246
Unmarried	309	12.4	34	18.1		23	13.0	
Divorced/separated	207	8.3	20	10.6		22	12.4	
Widower	5	0.2	0	0		0	0	
**Number of children under 18 years in the household**								
No children	566	25.5	54	34.0	0.044	41	29.7	0.745
One child	530	23.9	34	21.4		32	23.2	
2–4 children	1050	47.3	70	44.0		61	44.2	
Grand multiparity (five or more children)	72	3.2	<5	0.6		<5	2.9	
**Number of chronic somatic diseases**								
No chronic diseases/one chronic disease	1990	79.3	137	72.9	0.039	144	80.9	0.600
Two or more chronic diseases	521	20.7	51	27.1		34	19.1	
**Number of mental health diseases**								
No mental health diseases	2208	88.0	151	80.3	0.002	158	88.8	0.773
One or more mental health diseases	300	12.0	37	19.7		20	11.2	
**15D sum score**	0.936 (0.062)		0.916 (0.080)		0.008	0.937 (0.056)		0.702
**15D quartiles**								
Lowest quartile (<0.909)	460	24.2	53	37.6	<0.001	28	22.2	0.613
Other quartiles	1440	75.8	88	62.4		98	77.8	
**Mother’s educational status (during pregnancy)**								
Low 0–4 years	195	7.9	10	5.5	0.236	23	13.1	0.027
Intermediate 5–8 years	1379	56.1	96	53.0		100	57.1	
High ⩾ 9 years	886	36.0	75	41.1		52	29.7	
**Mother’s occupational status (during pregnancy)**								
No occupation	729	29.9	51	28.3	<0.001	52	29.7	0.003
Low social class	1085	44.5	107	59.4		59	33.7	
High social class	626	25.7	22	12.2		64	36.6	

Data are presented as number and percentage or mean and SD (standard deviation) of the population. *N* varies due to missing data. The *p*-value presents the statistical differences between two-parent family and the other family structures in terms of estimated variables, and were evaluated using Pearson’s chi-squared test or Fisher’s exact test, when appropriate, excluding 15D sum score, which was tested with Mann–Whitney *U*-test. The null hypothesis was that the estimated coefficients for the given family structure are not different from those estimated for the two-parent family offspring.

15D: 15-dimensional.

Table III shows the association between the offspring’s family structure at the age of 14 and HRQoL at the age of 46 years for females and males. The logistic models are presented as unadjusted models and models adjusted for offspring mothers’ factors during pregnancy (mothers’ educational and occupational status). Among both sexes, the offspring belonging to the ‘one parent not living at home/no information on father’ family structure subgroup had higher OR of belonging to the lowest 15D quartile in comparison with the ‘two-parent family’ offspring (adjusted OR 1.76, 95% CI 1.31–2.36, *p*<0.001 for females; adjusted OR 1.86, 95% CI 1.28–2.70, *p*=0.001 for males). There were no statistically significant associations between the ‘father or mother deceased’ subgroup and the lowest 15D quartile among the offspring.

**Table III.** Association between offspring’s family structure at the age of 14 years and belonging to the lowest quartile of health-related quality of life at the age of 46 years.

	**The lowest 15D quartile**	**Other 15D quartiles**	**Unadjusted**		**Adjusted** ^a^	
**Females**	*n* (%)	*n* (%)	OR (95% CI)	*p*	OR (95% CI)	*p*
One parent not living at home/No information on father	80 (34.0)	155 (66.0)	**1.65** (1.24–2.20)	0.001	**1.76** (1.31–2.36)	<0.001
Father or mother deceased	55 (29.4)	132 (70.6)	1.33 (0.96–1.85)	0.090	1.34 (0.96–1.87)	0.089
Two-parent family	586 (23.8)	1874 (76.2)	Ref.		Ref.	
**Males**	*n* (%)	*n* (%)	OR (95% CI)	*p*	OR (95% CI)	*p*
One parent not living at home/No information on father	53 (37.6)	88 (62.4)	**1.89** (1.32–2.69)	<0.001	**1.86** (1.28–2.70)	0.001
Father or mother deceased	28 (22.2)	98 (77.8)	0.89 (0.58–1.38)	0.610	0.94 (0.61–1.46)	0.788
Two-parent family	460 (24.2)	1440 (75.8)	Ref.		Ref.	

a Adjusted for confounding factors from the cohort members’ mothers’ pregnancy period: mothers’ educational and occupational status. Odds ratios (ORs; statistically significant values in bold), 95% confidence intervals (CIs) and *p*-values are presented as results, *p*-values less than 0.05 are considered statistically significant. Groups ‘Other 15D quartiles’ and ‘Two-parent family’ were used as the reference (Ref.).

15D: 15-dimensional.

**Figure 1.**
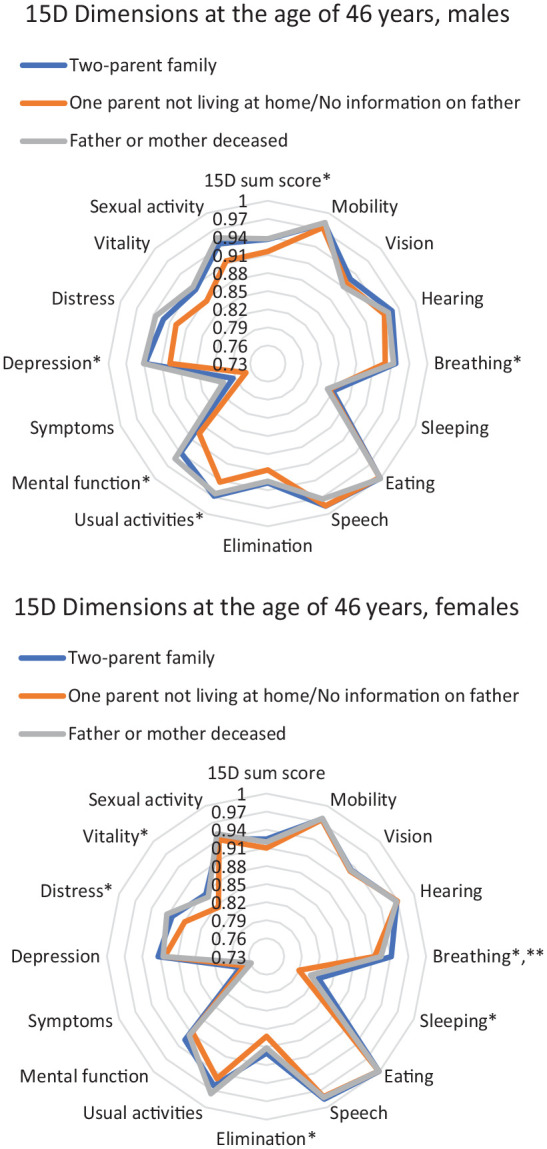
15D dimensions according to different childhood family structures at the age of 46 years, for females and males. 15D health-related quality of life 15 dimensions among the study population in different childhood family structures: ‘Two-parent family’ (dark blue), ‘one parent not living at home/no information on father’ (orange) and ‘father or mother deceased’ (grey). The closer the value is to the centre, the lower the dimension’s score. *p < 0.05 and the score is at least 0.015 units lower for the ‘one parent not living at home/no information on father’ subgroup and **p < 0.05 and the score is at least 0.015 units lower for the ‘father or mother deceased’ subgroup than for the ‘two-parent family’ subgroup. 15D: 15-dimensional.

Figure 1 show the offspring’s 15D dimensions at the age of 46 years according to different childhood family structures. The dimensions that scored at least 0.015 units lower among the female ‘one parent not living at home/no information on father’ offspring than among the female ‘two-parent family’ offspring were breathing, sleeping, elimination, distress and vitality (*p*<0.050). In turn, the dimensions that scored at least 0.015 units lower among the male ‘one parent not living at home/no information on father’ offspring than among the male ‘two-parent family’ offspring were 15D sum score, breathing, usual activities, mental function, and depression (*p*<0.050). Furthermore, the only dimension to score at least 0.015 units lower among the female ‘father or mother deceased’ offspring than among the female of ‘the two-parent family’ offspring was the breathing dimension (*p*<0.050).

## Discussion

In this prospective, longitudinal study of 6375 participants, we report the association between childhood family structure at the age of 14 years and HRQoL in midlife. We discovered that offspring living in a single-parent family (due to reasons other than parental death) in childhood were over one-and-a-half-times more likely to have significantly impaired HRQoL in midlife than the offspring of two-parent families. The estimates (adjusted for the following offspring mothers’ factors during pregnancy: mothers’ educational and occupational status) were similar for both sexes.

Our results align with previous reports showing that experiencing parental separation in childhood associates with lower quality of life in adulthood [14]. Vederhus et al. investigated the association between parental separation and adult quality of life in a retrospective and cross-sectional design, whereas in our study, we took a prospective and longitudinal approach, covering over 30 years of follow-up. Therefore, our results add to existing knowledge indicating the predictive role of potential parental separation in impaired HRQoL at midlife. We also observed that female offspring affected by parental death were more likely to have impaired HRQoL in adulthood than the offspring of two-parent families, but this finding did not reach statistical significance. This finding is partly in line with previous studies reporting that offspring affected by family-related stressful life events or adverse childhood experiences (including parental death) more often have impaired quality of life in adolescence [13] and adulthood [14] than those who have no such experiences.

Multifactorial mechanisms support our observations that offspring living in single-parent families in childhood (due to parental separation or parental death) have higher ORs of significantly impaired HRQoL in midlife than offspring living with two biological parents. First, it has been reported that half of the offspring of single-mother families (which was the most common family structure of single-parent families in the 1980s when the offspring were aged 14) [27] have no further contact with their fathers after parental divorce [28]. This potential lack of adult support has been strongly associated with impaired quality of life in adulthood [14]. However, it should be noted that some offspring may have a support network (e.g. grandparents), fulfilling the need for adequate support. Second, mental distress and social isolation have shown to mediate the association between parental separation and an adult’s impaired quality of life [14]. Even though these reports were based on cross-sectional data and did not evaluate HRQoL as the outcome, mental distress and social isolation may explain the detected family structure–HRQoL associations, particularly between the ‘one parent not living at home/no information on father’ group and impaired HRQoL.

In addition, compared with offspring living with two parents, offspring living with one parent or in other family constellations have reported lower quality of life [10]. It may be that impaired quality of life tracks from childhood to adulthood. Moreover, according to the existing literature, the odds of somatic and mental health morbidity later in life are higher among offspring who have been affected by parental separation or parental death in childhood [3,4,29]. For example, the higher use of psychoactive drugs among offspring of single-parent family compared with two-parent family offspring, a finding from our previous NFBC1966 study [3], might have impaired their HRQoL. Multimorbidity has also been associated with impaired HRQoL in midlife [30]. In the current study, we observed a higher proportion of multimorbidity among offspring of the ‘one parent not living at home/no information on father’ subgroup compared with offspring of the ‘two-parent family’ subgroup, which may partly explain the results.

The large birth cohort sample and the longitudinal approach from childhood to middle age are the major strengths of the present study. Furthermore, despite the over 30-year follow-up, the study had a substantially high response rate: 52%. There were significant differences between the participant and non-participant cohort members in the analysis of representativeness, for example, the participants were more often women and the proportion of mothers who were highly educated and had high occupational status was greater, which should be considered in interpreting our results. However, it is a well-recognized phenomenon in longitudinal surveys that lower levels of education and a lower income increase the probabilities of dropping out [31]. Using a valid instrument to measure HRQoL (15D) enabled us to evaluate it comprehensively. Overall, this is among the first longitudinal cohort studies to investigate the association between childhood family structure and HRQoL in adulthood. The data on childhood family structure were collected when the offspring were 14 years old. Unfortunately, we had no adequate data on the exact time point that parental separation or death had occurred. Similarly, we had no information on possible family reconstructions or exact information on siblings available, and their association with family dynamics and offspring’s HRQoL. These can be considered limitations of this study. It might be different living in a single-parent family today from how it was a few decades ago, and also divorce rates have increased since the family structure data collection point, and therefore generalizability of these results should be made with caution. On the other hand, it should be noted that even if the distribution of the family structure categories would change over time, its exogenous relationship with the outcome may not. In addition, all the data were ascertained through self-reports, which may have been influenced by subjective perceptions and social desirability. Moreover, it should be noted that some CIs for the ORs were slightly wide, thus the results should be verified in study populations with larger sample sizes.

## Conclusions

In conclusion, this large birth cohort study observed that belonging to the ‘one parent not living at home/no information on father’ family structure in childhood is associated with significantly impaired HRQoL in middle age among both sexes. These findings extend and strengthen the knowledge on the far-reaching associations of childhood family structure with individuals’ welfare. It would be beneficial for health care professionals and social workers to take patients’ whole lifespans into account when encountering them and dealing with their health problems, and society should support single-parent families in particular. Research on the associations between childhood family structure and the offspring’s later welfare and HRQoL should be continued, and future studies should extend the perspective to reconstructed families and the consequences of improved separation skills.

## Supplemental Material

sj-xlsx-1-sjp-10.1177_14034948241260765 – Supplemental material for Association between childhood family structure and health-related quality of life at middle age: A longitudinal study of Northern Finland Birth Cohort 1966 Supplemental material, sj-xlsx-1-sjp-10.1177_14034948241260765 for Association between childhood family structure and health-related quality of life at middle age: A longitudinal study of Northern Finland Birth Cohort 1966 by Heidi Varis, Eveliina Heikkala, Ilona Mikkola, Tanja Nordström, Anja Taanila, Sirkka Keinänen-kiukaanniemi and Maria Hagnäs in Scandinavian Journal of Public Health
